# Relationship between Initial Telomere Length, Initial Telomerase Activity, Age, and Replicative Capacity of Nucleus Pulposus Chondrocytes in Human Intervertebral Discs: What Is a Predictor of Replicative Potential?

**DOI:** 10.1371/journal.pone.0144177

**Published:** 2015-12-03

**Authors:** Jun-Seok Lee, Seo-Won Jeong, Sung-Wook Cho, Joon-Pyo Juhn, Ki-Won Kim

**Affiliations:** 1 Department of Orthopedic Surgery, Yeouido St. Mary’s Hospital, College of Medicine, The Catholic University of Korea, Seoul, Korea; 2 Department of Orthopedic Research, Medical Research Institute, Yeouido St. Mary’s Hospital, College of Medicine, The Catholic University of Korea, Seoul, Korea; Tulane University Health Sciences Center, UNITED STATES

## Abstract

There is evidence that telomere length (TL), telomerase activity (TA), and age are related to the replicative potential of human nucleus pulposus chondrocytes (NPCs). However, it has not yet been established if any of these factors can serve as predictors of the replicative potential of NPCs. To establish predictors of the replicative potential of NPCs, we evaluated potential relationships between replicative capacity of NPCs, initial TL (telomere length at the first passage), initial TA (telomerase activity at the first passage), and age. Nucleus pulposus specimens were obtained from 14 patients of various ages undergoing discectomy. NPCs were serially cultivated until the end of their replicative lifespans. Relationships among cumulative population doubling level (PDL), initial TL, initial TA, and age were analyzed. Initial TA was negatively correlated with age (r = -0.674, *P* = 0.008). However, no correlation between initial TL and age was observed. Cumulative PDL was also negatively correlated with age (r = -0.585, *P* = 0.028). Although the cumulative PDL appeared to increase with initial TL or initial TA, this trend was not statistically significant. In conclusion, age is the sole predictor of the replicative potential of human NPCs, and replicative potential decreases with age. Initial TL and initial TA are not predictors of replicative potential, and can serve only as reference values.

## Introduction

Back pain is a major health problem, and it is estimated that approximately two-thirds of the world’s population will experience lower back pain at some point in their lives [1, 2]. Disc degeneration has been reported in approximately 40% of back pain cases [3] and has a probable causal relationship with back pain [2, 4]. Current treatment approaches for degenerative intervertebral disc (IVD) diseases are restricted to symptomatic therapies, and cannot restore original structure and biological function. However, progress in tissue engineering and regenerative medicine has increased interest in developing biological therapeutic approaches capable of regenerating degenerative IVDs; cells alone or together with biomaterials have been implanted into the nucleus pulposus (NP) to both repopulate and stimulate native cells to produce a healthier extracellular matrix [2, 5, 6].

Telomeres are specialized nucleotide sequences at the ends of chromosomes and are comprised of tandem repeats of the sequence TTAGGG [7, 8]. Telomeres allow the ends of linear chromosomal DNA to be replicated completely without loss of terminal bases, and thereby preserve chromosome integrity by distinguishing natural chromosome ends from double-stranded DNA breaks [7]. Telomere length has been demonstrated to both reflect and limit the replicative lifespan of normal somatic cells [9]. Indeed, the replication of normal somatic cells is limited by telomere shortening, which proceeds incrementally with each round of cell division [10, 11], resulting in the loss of 50–200 terminal base pairs of the telomere in humans both *in vitro* and *in vivo* [12, 13]. With consecutive rounds of DNA replication, the telomeres become shorter (an end-replication problem) [7, 12, 14, 15]. However, telomerase, a ribonucleoprotein enzyme, can elongate telomeric repeats in the 5'-to-3' direction, thereby mitigating the end-replication problem [8, 15, 16]. Incomplete replication in the absence of telomerase results in progressive telomere shortening, which leads to the DNA damage response and replicative senescence [17]. Therefore, adequate telomere length and telomerase activity are necessary for replication of cells [8, 11].

Several recent studies have reported that reinsertion of autologous NP cells or stem cells delays degeneration in experimental models of disc degeneration [17–21]. In addition, injection of autologous NP cells has been performed in human with herniated discs [19]. Studies on growth factors and gene therapy have also reported promising results with respect to disc regeneration [22–26]. However, such studies should be performed based on a thorough understanding of the replicative potential of nucleus pulposus chondrocytes (NPCs). Indeed, if a study investigating a biological therapy for disc regeneration, such as cell-based therapy, growth factors administration, or gene therapy is performed using NPCs that no longer have normal replicative potential, it would be difficult to precisely determine their effect on cell proliferation and matrix synthesis. Therefore, for such biological therapies to be successful, it is necessary to either know or be able to predict the replicative potential of NPCs that are implanted, treated with growth factors, or subjected to gene therapy.

In a previous *in vitro* study on the mechanism of senescence of human NPCs, we showed that division of NPCs results in progressive telomere shortening and decreased telomerase activity [27]. We also observed a negative correlation between replicative potential of human NPCs and donor age [27]. However, this effect was not entirely clear due to the small sample size. Other recent studies reported that transfection of human NPCs with human telomerase reverse transcriptase (the catalytic subunit of telomerase) resulted in maintenance of telomere length and extended replicative potential [28, 29]. Together, these studies suggest that telomere length, telomerase activity, and age all contribute to the replicative potential of human NPCs. However, a reliable predictor of the replicative potential of NPCs has not yet been identified. Thus, we analyzed additional NPC preparations to clearly establish whether there is a relationship between telomere length, telomerase activity, age, and replicative potential.

We performed this study to evaluate telomere length dynamics and telomerase activity of long-term cultured NPCs from donors of various ages. Specifically, we sought to verify the relationships among initial telomere length (initial TL, telomere length at the first passage), initial telomerase activity (initial TA, telomerase activity at the first passage), age, and replicative capacity, and to determine whether any of these factors can serve as predictors of the replicative potential of human NPCs.

## Materials and Methods

### Subjects

NP specimens were obtained from 14 patients of different ages (mean: 53 years, range: 32–72 years) who underwent open discectomy for a symptomatic herniated NP. An attempt was made by the operating surgeons (J.S.L., K.W.K.) to carefully obtain tissue from the central aspect of the disc to optimize harvest of only NP tissue. Extruded or sequestrated NP fragments were removed. Then, the NP specimens, which remained in the central part of the IVD, were pooled in a 50 mL conical tube containing 30 mL of Dulbecco’s Modified Eagle’s medium (DMEM, GIBCO-BRL, Grand Island, NY, USA) and transferred immediately to a cell culture laboratory. NP specimens were classified by decade into five age groups as follows: 30s (30 to 39 years, n = 3), 40s (n = 3), 50s (n = 3), 60s (n = 3), and 70s (n = 2). NP specimens were also grouped according to a grading system for IVD degeneration, which was based on preoperative magnetic resonance images (MRI) [30]. Grades III and IV were included in our study to minimize the effect of degeneration grades on the replicative capacity, telomere length, and telomerase activity of human NPCs. Six specimens had grade III degeneration and eight had grade IV degeneration. All human NP specimens were obtained with the written informed consent of the patients. The data were recorded and analyzed anonymously. All of the consent procedure and experimental protocols were approved by the Institutional Review Board of the Catholic University of Korea (SC13SISI0145).

### Isolation and culture of human NPCs

NP tissues were identified by their macroscopic morphology and were carefully separated from any obvious granulation tissue, cartilaginous endplates, or annulus. NPCs were isolated as described in our previous studyies [31, 32]. Briefly, whole NP tissue was minced with a scalpel into pieces of approximately 0.3 cc in volume. The NP tissue was then digested with 2 mg/mL of collagenase type II (GIBCO-BRL) in DMEM for 16 hours at 37°C. The digested NP tissue was washed twice with DMEM, and NPCs were subsequently isolated. Cells were maintained in complete medium (DMEM supplemented with 10% fetal bovine serum, 1% penicillin-streptomycin [GIBCO-BRL]) at 37°C in a humidified atmosphere containing 5% CO_2_ until they grew to confluence. Cells were fed by complete replacement of medium every 3 days. When the primary cultures reached confluence, adherent cells were detached using 0.25% trypsin EDTA solution (GIBCO-BRL) and seeded at a density of 1 × 10^5^ cells/mL in 100-mm culture dishes containing complete medium. Upon reaching confluence, cells were again trypsinized and passaged sequentially in the same manner until they reached the end of their replicative lifespan. Throughout the experiment, human NPCs were cultivated under standard culture conditions.

### Replicative capacity measured by cumulative population doubling level (PDL)

At each subcultivation, confluent NPCs were trypsinized, counted, and reseeded at a density of 1 × 10^5^ cells per dish. The cumulative PDL at each subcultivation was calculated from cell counts using the following equation [33]: PD = [log_10_(*N*_H_)–log_10_(*N*_I_)] / log_10_(2), where *N*_H_ is the number of harvested cells and *N*_I_ is the number of seeded cells. An increase in population doubling (PD) was calculated and added to the previous PD to determine the cumulative PDL. The end of the replicative lifespan was defined as the failure of the population to double after 4 weeks in culture with 3 weeks of consecutive refeeding as described by Cristofalo et al. [33]. At each subcultivation, measurement of PDL was repeated twice and the mean was calculated.

### Telomere length assay

Telomere length assay was performed as described in our previous studies [31, 32]. Briefly, at every other passage, NPCs were harvested and genomic DNA was extracted using a genomic DNA purification kit (Gentra, Minneapolis, MN, USA). Genomic DNA (3 μg) was digested with the restriction enzymes *Rsa* I and *Hinf* I at 37°C for 16 hours. Subsequently, the digested DNA was electrophoresed on 0.8% agarose gels at 50 V for 2.5 hours. After electrophoresis, gels were depurinated in 0.25 *M* HCl for 30 minutes and denatured in 0.4 *M* NaOH and 1.5 *M* NaCl for 30 minutes, after which the DNA was transferred to a positively charged nylon membrane (Hybond-N, Roche, Mannheim, Germany) by capillary transfer. Membranes were prehybridized for one hour at 42°C with digoxigenin (DIG) Easy Hyb and then hybridized in solution by adding 1 μL of telomere probe per 5 mL of fresh prewarmed DIG Easy Hyb. Subsequently, the membrane containing the DNA fragments linked to telomere probes was incubated with a digoxigenin-specific antibody covalently coupled to alkaline phosphatase. Results were visualized using alkaline phosphatase metabolizing CDP-Star, a highly sensitive chemiluminescent substrate. Subsequently, the membrane was exposed to Hyperfilm (GE Healthcare, Buckinghamshire, UK). Mean telomere length was calculated using the formula: L = ∑(*OD*_*i*_) / ∑(*OD*_*i*_ / *L*_*i*_), where *OD*_*i*_ is the integrated signal intensity and *L*_*i*_ is the DNA length at position *i*. The mean rate of telomere shortening per PD was assessed during the period from the first passage to the last passage that telomere length was checked.

### Telomerase activity assay

Telomerase activity was evaluated by Telomeric Repeat Amplification Protocol (TRAP) assay using the quantitative TeloTAGGG Telomerase PCR ELISA^PLUS^ kit (Roche-Applied Science, Mannheim, Germany). Briefly, NPCs (1x10^5^) were lysed in 200 μl of ice-cold lysis reagent and incubated on ice for 30 min. Then the lysate was centrifuged at 16,000 ×*g* for 20 min at 4°C. The supernatant was gently collected and the protein concentration in the supernatant was measured with the BCA protein assay (Pierce Biotechnology, Rockford, IL, USA) using BSA as a standard. The total volume of the PCR reaction mixture was 50 μl, comprising 25 μl of reaction mixture, 5 μl of internal standard (IS), 10 μg of sample, negative control, control templates (TS8), and nuclease-free water. All samples were run in duplicate. Lysis buffer was used as a negative control and the control template was used directly from the kit. PCR mixtures were transferred to a thermal cycler and primer elongation was performed for 30 min at 25°C. The PCR cycling parameters were as follows: 94°C for 5 min followed by 30 cycles of 94°C for 30 s, 50°C for 30 s, and 72°C for 90 s. For each of the samples, 10 μl of denaturation reagent was pipetted into separate reaction tubes, followed by addition of 2.5 μl of amplification product and incubation at 15–25°C for 10 min. Then, 100 μl of hybridization buffer T was added to one tube and 100 μl of hybridization buffer IS was added to the other tube. As described in the TeloTAGGG Telomerase PCR ELISA^PLUS^ instruction manual, 100 μl of each mixture was transferred into each well of streptavidin-coated MTP modules supplied with the kit. The MTP modules were incubated at 37°C for 2 hours on a shaker at 300 rpm. Hybridization solution was removed and each well was washed with washing buffer. Then, 100 μl of Anti-DIG-HRP working solution was added to each well followed by an incubation at 15–25°C for 30 min at 300 rpm. Each well was washed with washing buffer, the washing buffer was removed, and 100 μl of TMB substrate solution was added to each well followed by a 30 min incubation at 15–25°C at 300 rpm. Without removing the reaction mixture, 100 μl of stop reagent was added to each well to stop color development. The absorbance of the samples was measured at 450 nm using a microplate spectrophotometer (PowerWave XS; BioTek, Winooski, VT, USA) within 30 min after adding the stop reagent. We subtracted the mean of the absorbance readings of the negative controls form absorbance readings of the samples (A_S_–A_S,0_). Samples were to be considered as telomerase-positive if the difference in absorbance (△A) was higher than the two-fold background activity (background activity = value of negative control or heat-treated sample). The level of telomerase activity in a sample was determined by comparing the signal from the sample to the signal obtained using a known amount of a control template (TS8). The control template (control template, low) provided with the TeloTAGGG Telomerase PCR ELISA^PLUS^ was ready to use solutions that contained TS8 at a concentration of 0.001 amol/mL. The control template used was identical to a telomerase elongation product with 8 telomeric repeats. Finally, relative telomerase activities (RTAs) within different samples were obtained using the following formula: RTA = [(A_S_–A_S,0_ / A_S,IS_) / (A_TS8_–A_TS8,0_ / A_TS8,IS_)] x 100, where A_S_ is the absorbance of sample, A_S,0_ is the absorbance of heat-treated sample, A_S,IS_ is the absorbance of internal standard (IS) of the sample, A_TS8_ is the absorbance of control template (TS8), A_TS8,0_ is the absorbance of Lysis buffer, and A_TS8,IS_ is the absorbance of internal standard (IS) of the control template (TS8).

Telomere length and telomerase activity were assessed at passages 1, 3, and 5. Telomere length and telomerase activity were also assessed at passages 7, 9, and 11 when the amount of NPCs, except for the NPCs used in each subculture, was sufficient for the assay. All experiments were performed twice to ensure consistency.

### Statistical analysis

The significance of differences in the means of non-parametric variables between groups was analyzed using the Mann-Whitney U test. Correlations among variables were analyzed by Spearman’s rho test. All data are presented as means (±SDs). *P*-values < 0.05 were considered significant.

## Results

The characteristics of nucleus pulposus tissues obtained from patients as well as cumulative PDL, initial TL, and initial TA of NPCs are shown in Table 1.

**Table 1:** Summary of the characteristics of nucleus pulposus tissues obtained from patients: cumulative population doubling level, initial telomere length, and initial telomerase activity of nucleus pulposus chondrocytes.

Age/Sex	Disc level	Deg. grade	Maximum cPDL	Initial TL (kb)	Initial TA (RTAs)
39/M	L34	IV	34.0	22.0 (0.64)	31.5 (2.40)
32/M	L45	III	29.5	13.4 (0.23)	29.9 (0.84)
37/M	L5S1	III	25.9	11.1 (0.15)	24.3 (1.55)
44/M	L5S1	III	20.7	16.0 (0.45)	15.9 (1.83)
44/F	L45	III	20.9	14.2 (0.18)	15.1 (1.02)
41/M	L45	III	23.1	11.0 (0.15)	16.3 (0.84)
58/M	L23	IV	30.1	10.3 (0.12)	14.1 (1.37)
52/M	L5S1	IV	27.0	12.6 (0.62)	14.4 (3.38)
58/F	L45	IV	29.5	17.7 (0.35)	10.2 (1.24)
66/M	L45	IV	16.4	7.0 (0.38)	13.1 (2.77)
63/M	L5S1	IV	17.0	12.6 (0.12)	17.0 (2.77)
65/F	L45	III	22.1	19.7 (0.28)	9.9 (1.04)
72/M	L45	IV	20.8	11.0 (0.32)	9.3 (0.24)
71/F	L45	IV	17.3	18.1 (0.20)	22.3 (0.62)

M, male; F, female; Deg. grade, degeneration grade of intervertebral disc; cPDL, cumulative population doubling level; Initial TL, telomere length of nucleus pulposus chondrocytes at passage 1; Initial TA, telomerase activity of nucleus pulposus chondrocytes at passage 1; kb, kilobases; RTAs, relative telomerase activities. Data of initial TL and TA are mean (SD) from two assays.

### Replicative capacity measured by cumulative PDL

**Fig 1:**
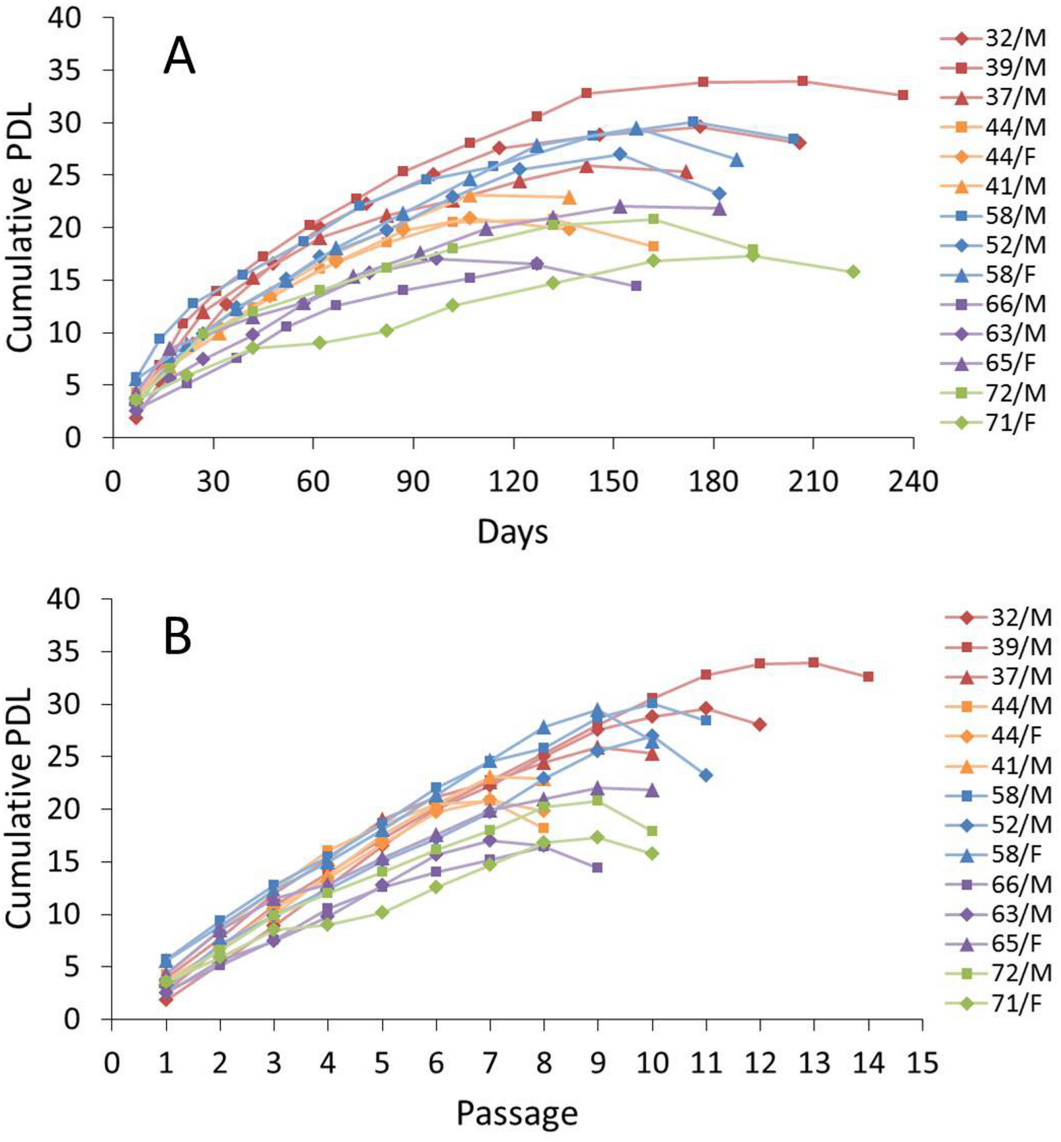
Long-term growth curves of nucleus pulposus chondrocytes (NPCs) obtained from donors of various ages. Plots of cumulative population doubling levels (PDLs) against time in culture (A) and passage number (B). The cumulative PDLs of NPCs from the young group (patients in their 30s, 40s, and 50s) were relatively higher than those from the old group (patients in their 60s and 70s). Plots of cumulative PDLs against time in culture showed a steep curve in the early passages and a flattening with increasing time. The early steepness was longer in cultures from the young group than in cultures from the old group. Plots of cumulative PDLs against passage number showed a reverse slope at the end, indicating a decrease in cumulative PDL in the last passage just after the maximum cumulative PDL. Cellular death presumably account for these population drops.

All of the NPCs examined exhibited a finite replicative capacity. Plotting the cumulative PDL of the NPCs at each subcultivation against the total time in culture showed that in all cases, the resulting line connecting the points changed from an early steep slope to a gentle slope with continued passaging. The growth curve also showed a reverse slope at the end, indicating a decrease in cumulative PDL in the last passage just after the maximum cumulative PDL. Cellular death may account for these population drops (Fig 1). The mean cumulative PDL was 23.9 (±5.5) and the mean cumulative PDL of the NPCs before reaching the limits of their replicative lifespans obtained from patients in their 30s, 40s, 50s, 60s, and 70s was 29.8 (±4.1), 21.6 (±1.3), 28.99 (±1.6), 18.5 (±3.1), and 19.1 (±2.5), respectively. NPCs derived from patients in their 30s, 40s, and 50s exhibited similar replicative potentials, while the replicative potentials of NPCs derived from patients in their 60s and older were significantly lower; the mean cumulative PDL was 26.7 (±4.5) for the young group (30s, 40s, and 50s) versus 18.7 (±2.6) for the old group (60s and 70s) (*P* = 0.007). There was no significant difference in cumulative PDL with respect to grade of degeneration. Specifically, the mean cumulative PDL was 24.5 (±5.0) for grade III and 23.5 (±6.2) for grade IV (*P* = 0.755).

### Telomere length dynamics

**Fig 2:**
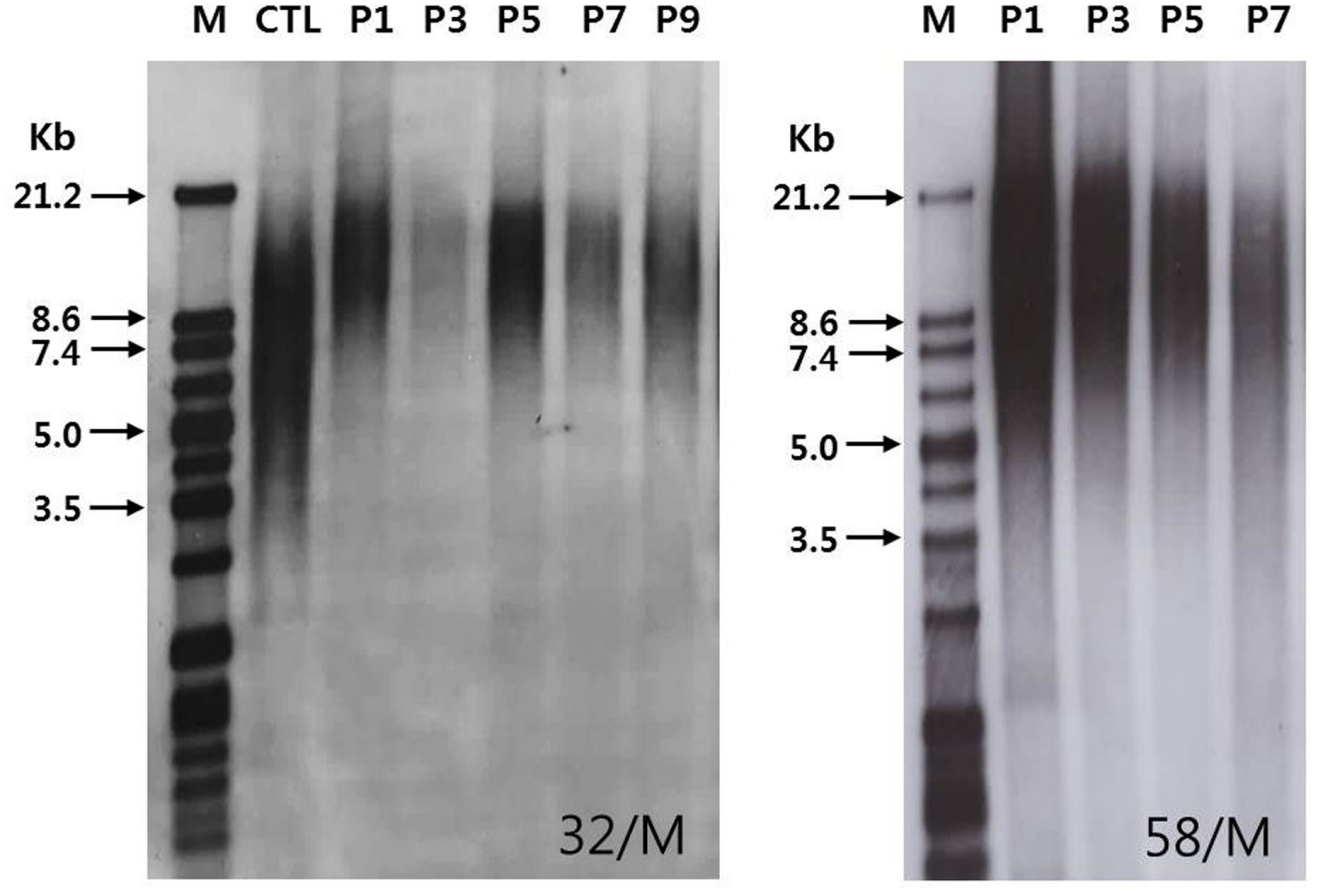
Southern blot analysis of telomere lengths of nucleus pulposus chondrocytes derived from two donors of different ages during multiple passages. There was a steady decline in telomere length with advancing passage number, irrespective of age (Kb, kilobases; M, marker; CTL, control; P, passage).

**Fig 3:**
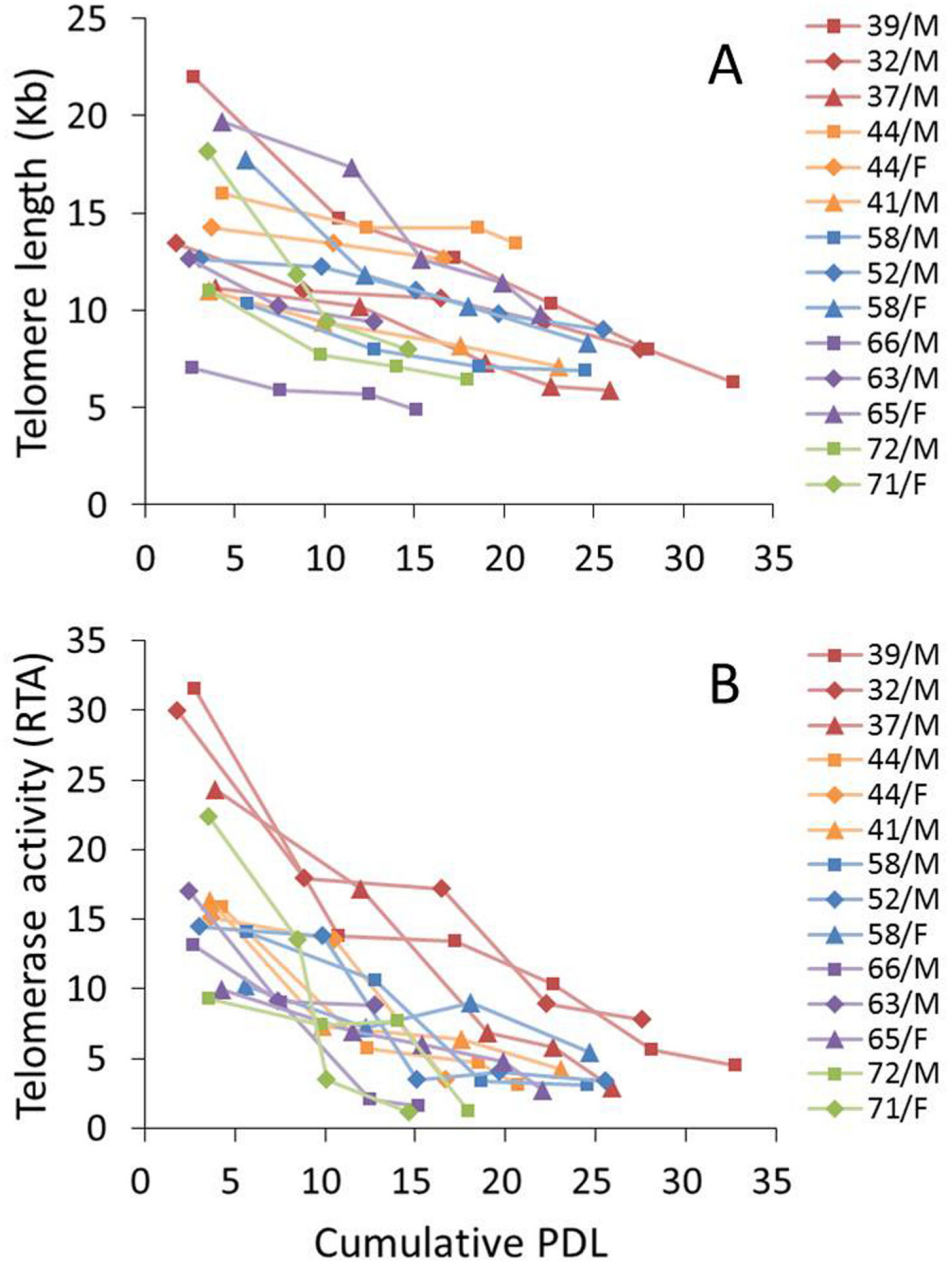
Changes in telomere length and telomerase activity of nucleus pulposus chondrocytes (NPCs) during long-term culture. Both (A) telomere length and (B) telomerase activity of NPCs exhibited a steady decline with advancing population doubling. Telomere lengths are provided in kilobases (kb) and the level of telomerase activity as relative telomerase activity (RTA).

Southern blot analysis was used to determine the telomere length of NPCs obtained from donors of different ages over the observation period (Fig 2). The initial TL was an average of 14.1 kb (range and SD: 7.0–22.0 kb, ±4.2 kb) and declined with PD, irrespective of age (Fig 3A). The average rate of telomere shortening was heterogeneous between the different NPC strains, ranging from 0.12 to 0.91 kb/PD (Mean ±SD, 0.33 ±0.2 kb/PD). Mean initial TLs of NPCs obtained from patients in their 30s, 40s, 50s, 60s, and 70s were 15.5 (±5.7), 13.7 (±2.5), 13.5 (±3.8), 13.1 (±6.4), and 14.6 (±5.0) kb, respectively. There was no significant difference in mean initial TLs between the young and old group; the mean initial TL was 14.3 (±3.8) kb in the young group (30s, 40s, and 50s) and 13.7 (±5.2) kb in the old group (60s and 70s) (*P* = 0.898). There was also no significant difference in mean initial TLs between degeneration grades; the mean initial TL was 15.7 (±4.5) kb for grade III and 12.8 (±3.7) kb for grade IV (*P* = 0.228).

### Telomerase activity

Levels of telomerase activity in human NPCs are presented as relative telomerase activities (RTAs). The telomerase activity of the positive control (the control template, low) was 0.205. The mean initial TA was 17.4 (range and SD: 9.3–31.5, ±7.1) and declined with PD, irrespective of age (Fig 3B). The mean initial TAs of NPCs obtained from patients in their 30s, 40s, 50s, 60s, and 70s were 28.6 (±3.8), 15.8 (±0.6), 12.9 (±2.3), 13.3 (±3.6), and 15.8 (±9.2), respectively. The mean initial TA of NPCs from patients in their 30s tended to be higher than those from the other patients. However, there was no significant difference in mean initial TAs between the young and old group; mean initial TA was 19.1 (±7.6) for the young group (30s, 40s, and 50s) and 14.3 (±5.4) for the old group (60s and 70s) (*P* = 0.24). There was also no significant difference in mean initial TA between degeneration grades; the mean initial TA was 18.8 (±7.7) for grade III and 16.3 (±6.8) for grade IV (*P* = 0.414).

### Relationship between replicative capacity of NPCs, initial TL, initial TA, and age

**Fig 4:**
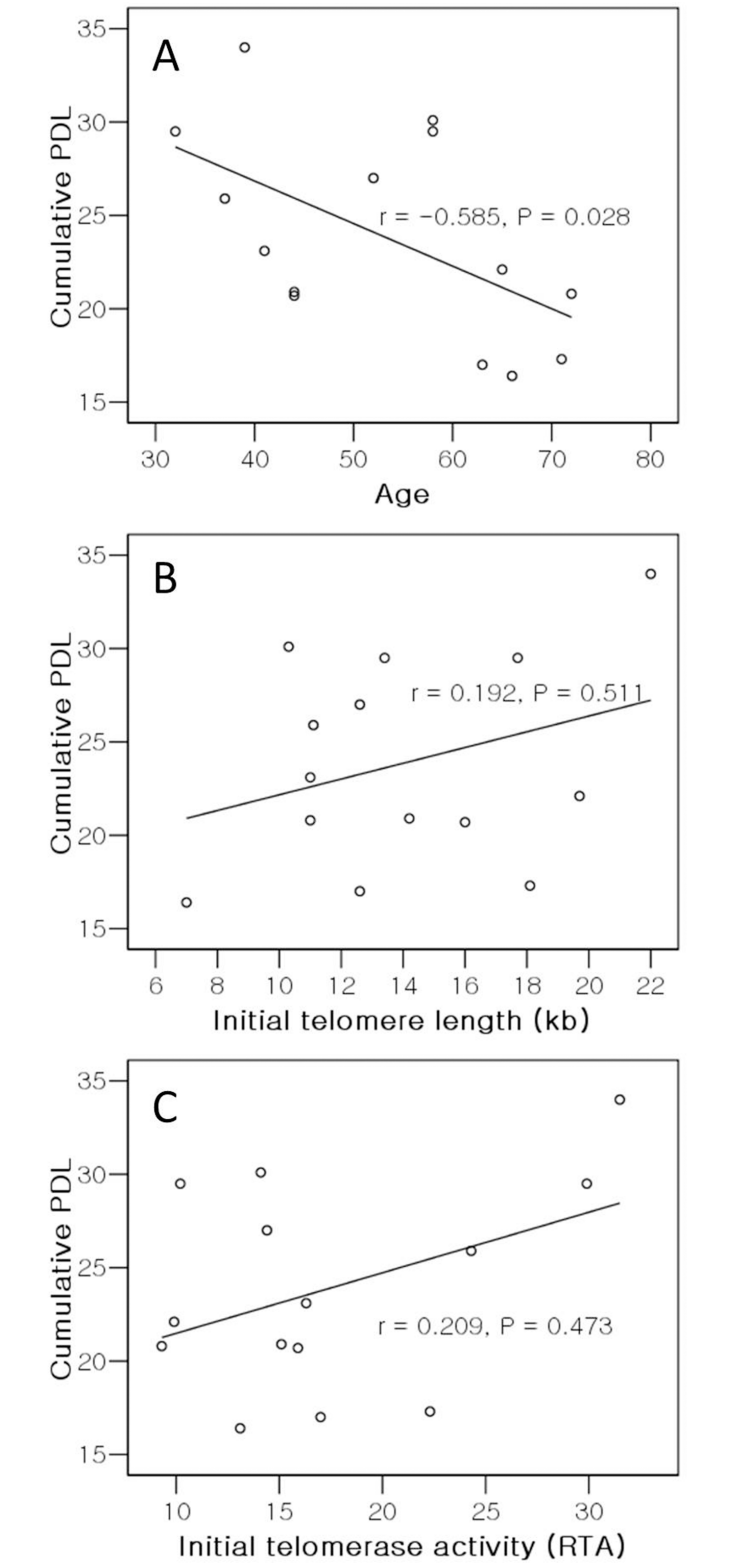
Correlation analysis of cumulative population doubling level (PDL) of nucleus pulposus chondrocytes versus (A) donor age, (B) initial telomere length, and (C) initial telomerase activity. Telomere length and telomerase activity at the first passage are indicated as initial telomere length and initial telomerase activity. Telomere lengths are provided in kilobases (kb) and the level of telomerase activity as relative telomerase activity (RTA).

**Fig 5:**
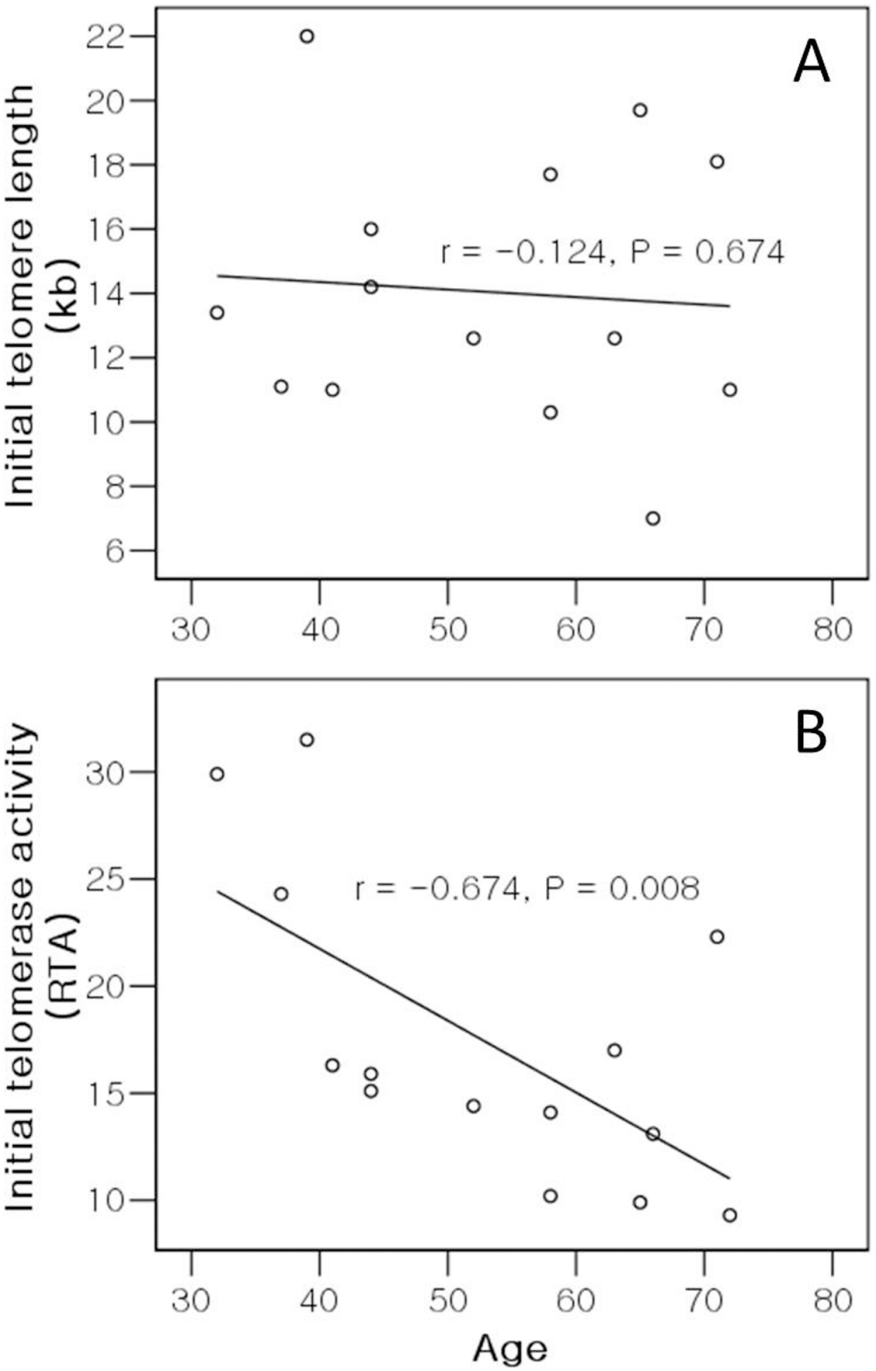
Correlation analysis of donor age versus (A) initial telomere length and (B) initial telomerase activity of nucleus pulposus chondrocytes. Telomere length and telomerase activity at the first passage are indicated by initial telomere length and initial telomerase activity. Telomere lengths are indicated in kilobases (kb) and the level of telomerase activity as relative telomerase activity (RTA).

There was a significant negative correlation between cumulative PDL and age (r = -0.585, *P* = 0.028; Fig 4A), indicating that the replicative capacity of NPCs decreased with donor age. Although the cumulative PDL appeared to increase with initial TL or initial TA, there was no correlation between cumulative PDL and initial TL (r = 0.192, *P* = 0.511; Fig 4B), nor between cumulative PDL and initial TA (r = 0.209, *P* = 0.473; Fig 4C); initial TL and TA did not affect the replicative capacity of NPCs *in vitro*. No correlation between initial TL and age was observed (r = -0.124, *P* = 0.674; Fig 5A), whereas a significant negative correlation between initial TA and age was observed, indicating that initial TA decreased with donor age (r = -0.674, *P* = 0.008; Fig 5B). However, the correlation between telomerase activity and age did not continue during passage; no correlation between age and either telomerase activity at passage 3 (r = -0.393, *P* = 0.054) or telomerase activity at passage 5 (r = -0.362, *P* = 0.077) was observed. Additionally, there was no correlation between age and the mean rate of telomere shortening (r = 0.313, *P* = 0.276), between initial TL and the mean rate of telomere shortening (r = 0.504, *P* = 0.066), between initial TA and the mean rate of telomere shortening (r = 0.077, *P* = 0.794), between cumulative PDL and the mean rate of telomere shortening (r = 0.106, *P* = 0.719), nor between initial TL and initial TA (r = 0.262, *P* = 0.365).

## Discussion

The present study clearly demonstrated that donor age is the sole predictor of the replicative potential of human NPCs and that the replicative potential of human NPCs decreases with increasing age. This age-dependent decrease in replicative potential of human NPCs is in accordance with the results of other studies that used cells from different sources, including vascular smooth muscle cells [34], peritoneal mesothelial cells [35], adrenocortical cells [36], and peripheral blood mononuclear cells [37], although another previous study failed to find a correlation between the replicative potential of skin fibroblasts and donor age [33].

Telomere length is used to deduce both the proliferation history and replicative potential of cells. Short telomeres are often taken as prima facie evidence that the associated cells have gone through many rounds of replication and thus have a limited future replicative potential [8]. Telomerase is capable of synthesizing terminal telomere repeats and extending the length of the telomere, thus compensating for the telomere shortening associated with cellular division [8]. Previous *in vitro* studies reported a positive correlation between telomere length and proliferative potential in fibroblasts [11] as well as peripheral blood mononuclear cells [37]. For these reasons, we expected that initial TL or initial TA would be a predictor of the replicative potential of human NPCs. However, according to our data, although the replicative potential of NPCs tended to increase with initial TL or initial TA, this association was not statistically significant. Thus, our results suggest that long telomeres or adequate telomerase activity do not always guarantee that human NPCs will have a higher replicative potential *in vitro*. These results also support findings from previous reports that telomere length can be short not only due to replication, but also due to other factors such as oxidative stress [38] and donor health [39].

Age-dependent telomere shortening has been demonstrated in different self-renewing human cells such as fibroblasts, peripheral blood monocytes, endothelial cells, and others [10, 36, 37]. However, in the present *in vitro* study of NPCs from human IVD tissue, no correlation between initial TL and age was observed. Additionally, it is worth noting that initial TL was highly variable among subjects within the same decade. This variation in telomere length may be due to differences in genetic determination [40], early life experiences and growth [41], and/or exposure to stress throughout an individual’s life [42]. However, at least in the case of NPCs, a possible explanation for the variation in telomere length may be related to the origin of NPCs. We previously characterized the migration of NPCs, and found that NPCs originate and migrate from vertebral cartilage endplates to NP lesions [43]. Furthermore, because NPCs divide and proliferate to fill the space created after apoptosis of notochordal NP cells during migration from vertebral cartilage endplates to NP lesions [44], it is not at all surprising that NPCs that migrate a short distance would have a lower proliferative history than NPCs that have migrated a longer distance. Indeed, such a scenario would generate a different number of cycles of mitosis during the migration of each NPC, thus explaining the differences in telomere lengths of NPCs from each other. Therefore, the variation in telomere length of NPCs observed in this study may be evidence of NPC migration. The variation in telomere length could also be explained by differences in the time at which NPCs start to migrate from vertebral cartilage endplates to NP lesions, as well as the difference in age of each individual with respect to the appearance of NPCs after apoptosis of notochordal cells in NP lesions. However, interpretation of these results is difficult because numerous other unknown factors may also affect telomere length.

We clearly demonstrated that NPCs have a detectable initial TA that is independent of telomere length but that decreases with age. This negative correlation between initial TA and donor age is consistent with findings reported for chondrocytes in articular cartilage [45]. Indeed, previous studies have provided evidence of the telomere length-independent functions of telomerase, which appear to promote cell survival and stress resistance [46, 47]. The telomere-independent and age-dependent decline in the initial TA of NPCs suggests that the survival capacity and stress resistance of NPCs decrease with age. In addition, there was a steady decline in both telomere length and telomerase activity of NPCs with increasing PD, irrespective of patient age, suggesting that telomerase does not counteract telomere shortening significantly during *in vitro* expansion of NPCs.

The results of the present study shed light on the replicative lifespan, telomere length, and telomerase activity of human NPCs obtained from donors of different ages grouped by decade. NPCs derived from patients in their 30s, 40s, and 50s had similar replicative potentials, which decreased significantly with age after the sixth decade of life; the mean cumulative PDL started to decrease significantly in patients aged 60 and older. Considering this result, young individuals (under 60 years) may be suitable candidates for cell-based therapies such as autologous NPC implantation to treat degenerative disc disease. However, there were no differences in telomere length among patients with respect to decade of life. In particular, the rate of telomere shortening was heterogeneous even among individuals of the same decade. Specifically, the rate of telomere shortening ranged from 0.12 to 0.91 kb/PD, which is a slightly larger range than the 0.18 to 0.21 kb/PD reported previously for NPCs [48]. However, considering that the previous study assessed the rate of telomere shortening using only two samples of non-degenerated IVD tissues obtained from one donor, a 37-year-old male, our results may be more reliable.

In the present study, the degeneration grade of the IVDs could only partially be controlled. IVDs used in this study were of degeneration grades III or IV. In practice, no NP specimens with grade I, II, or V degeneration could be obtained because tissues were sampled from patients with herniated discs, which generally do not occur in individuals with normal to near-normal discs (grade I or II) or with total collapse of the disc space (grade V). As a result, we were unable to identify any differences in replicative lifespan, telomere length, or telomerase activity in NPCs derived from grade III and grade IV IVDs. Thus, as we reported previously [31], it appears that degeneration grades, which are arbitrary assigned by MRI findings, may not directly reflect replicative capacity, changes in telomere length, or telomerase activity. The use of only two degeneration grades in this study may have also affected our statistical results. Additionally, in the present study, we did not use any markers for the NPCs. NPCs were identified by their macroscopic morphology. The morphology of NPCs changed during long-term culture. The initially isolated human NPCs had a round shape, consistent with a chondrogenic phenotype. After primary culture and subculture, NPCs became spindle-shaped, similar to fibrotic cells (data not shown).

Our study had several limitations. Firstly, the sample size was relatively small, which made it difficult to compare data among decades. Furthermore, the power of the correlation coefficient was relatively low, approximately 0.6–0.8. Thus, further studies with larger numbers of subjects are required. Secondly, we did not assess telomere lengths for all passages during the culture, because our aim was to determine whether initial TL or initial TA was a predictor of the replicative potential of NPCs or not, and to verify the pattern of change of telomere length and telomerase activity in culture. Thus, the shortest telomere length in the final passage was not assessed. Indeed, the length of the shortest telomere in cells, rather than the average telomere length, determines replicative senescence onset [49]. However, in the present study, we focused mainly on the relationship among initial TL, initial TA, age, and the replicative potential of NPCs. Additionally, in our previous *in vivo* study, we demonstrated age-dependent telomere shortening in human NPCs [31]. On the contrary, in the present *in vitro* study on human NPCs, no correlation between initial TL and age was observed. Thus, the results of this *in vitro* study may not be applicable *in vivo*. Finally, no healthy IVD controls were included in the present study. We carried out our experiments using only NPCs obtained from degenerative IVDs. However, if a study investigating biological therapies for disc regeneration, such as cell-based therapy, is performed using autologous NPCs, a degenerative IVD would be the source for autologous NPCs because the harvest of NPCs from a healthy IVD can result in degeneration of the healthy IVD [50]. Additionally, the targets of growth factor administration or gene therapy for disc regeneration are also degenerative IVDs. Thus, our data may be what is actually needed.

Despite these limitations, the present *in vitro* study demonstrated that, at least in the case of human NPCs, donor age is the sole predictor of replicative potential and that replicative potential decreases with age. Our results also indicated that initial TL and TA are not predictors of the replicative potential of human NPCs. Thus, initial TL and TA should be used only as reference values for replicative potential when studying human NPCs in the context of developing biological therapies to treat IVD degeneration.

## Supporting Information

S1 Table Cumulative population doubling levels of nucleus pulposus chondrocytes obtained from study subjects. (DOCX)

S2 Table Telomere length and telomerase activity of nucleus pulposus chondrocytes obtained from study subjects. (DOCX)
