# Supplementary material for: Relationship between Initial Telomere Length, Initial Telomerase Activity, Age, and Replicative Capacity of Nucleus Pulposus Chondrocytes in Human Intervertebral Discs: What Is a Predictor of Replicative Potential?
Source: PLoS One. 2015 Dec 3;10(12):e0144177. doi: 10.1371/journal.pone.0144177 (PMC4669191; doi:10.1371/journal.pone.0144177)
Supplement: S1 Table — (DOCX) [file pone.0144177.s001.docx]

**S1 Table. Cumulative population doubling levels of nucleus pulposus chondrocytes obtained from study subjects.**

| **Age/Sex** |  | **P1** | **P2** | **P3** | **P4** | **P5** | **P6** | **P7** | **P8** | **P9** | **P10** | **P11** | **P12** | **P13** | **P14** |
| --- | --- | --- | --- | --- | --- | --- | --- | --- | --- | --- | --- | --- | --- | --- | --- |
| **39/M** | Days | 7 | 14 | 21 | 31 | 45 | 59 | 73 | 87 | 107 | 127 | 142 | 177 | 207 | 237 |
|  | cPDL | 2.7 | 6.8 | 10.8 | 13.9 | 17.2 | 20.2 | 22.7 | 25.4 | 28.1 | 30.5 | 32.8 | 33.8 | 34.0 | 32.5 |
| **32/M** | Days | 7 | 14 | 24 | 34 | 48 | 62 | 76 | 96 | 116 | 146 | 176 | 206 |  |  |
|  | cPDL | 1.8 | 5.3 | 8.9 | 12.7 | 16.5 | 20.0 | 22.3 | 25.0 | 27.6 | 28.8 | 29.5 | 28.0 |  |  |
| **37/M** | Days | 7 | 17 | 27 | 42 | 62 | 82 | 102 | 122 | 142 | 172 |  |  |  |  |
|  | cPDL | 3.9 | 7.7 | 12.0 | 15.3 | 19.0 | 21.2 | 22.7 | 24.5 | 25.9 | 25.3 |  |  |  |  |
| **44/M** | Days | 7 | 22 | 42 | 62 | 82 | 102 | 132 | 162 |  |  |  |  |  |  |
|  | cPDL | 4.3 | 8.5 | 12.4 | 16.0 | 18.6 | 20.5 | 20.7 | 18.2 |  |  |  |  |  |  |
| **44/F** | Days | 7 | 17 | 32 | 47 | 67 | 87 | 107 | 137 |  |  |  |  |  |  |
|  | cPDL | 3.7 | 6.7 | 10.5 | 13.4 | 16.7 | 19.7 | 20.9 | 19.8 |  |  |  |  |  |  |
| **41/M** | Days | 7 | 17 | 32 | 47 | 67 | 87 | 107 | 137 |  |  |  |  |  |  |
|  | cPDL | 3.6 | 6.7 | 9.9 | 13.8 | 17.6 | 20.5 | 23.1 | 22.9 |  |  |  |  |  |  |
| **58/M** | Days | 7 | 14 | 24 | 39 | 57 | 74 | 94 | 114 | 144 | 174 | 204 |  |  |  |
|  | cPDL | 5.7 | 9.4 | 12.8 | 15.5 | 18.7 | 22.1 | 24.5 | 25.8 | 28.7 | 30.1 | 28.4 |  |  |  |
| **52/M** | Days | 7 | 17 | 27 | 37 | 52 | 62 | 82 | 102 | 122 | 152 | 182 |  |  |  |
|  | cPDL | 3.1 | 7.1 | 9.9 | 12.4 | 15.1 | 17.2 | 19.7 | 22.9 | 25.5 | 27.0 | 23.2 |  |  |  |
| **58/F** | Days | 7 | 22 | 37 | 52 | 67 | 87 | 107 | 127 | 157 | 187 |  |  |  |  |
|  | cPDL | 5.6 | 8.9 | 12.3 | 15.0 | 18.1 | 21.4 | 24.7 | 27.9 | 29.5 | 26.5 |  |  |  |  |
| **66/M** | Days | 7 | 22 | 37 | 52 | 67 | 87 | 107 | 127 | 157 |  |  |  |  |  |
|  | cPDL | 2.7 | 5.1 | 7.6 | 10.5 | 12.5 | 14.0 | 15.2 | 16.4 | 14.4 |  |  |  |  |  |
| **63/M** | Days | 7 | 17 | 27 | 42 | 57 | 77 | 97 | 127 |  |  |  |  |  |  |
|  | cPDL | 2.5 | 5.6 | 7.4 | 9.8 | 12.8 | 15.7 | 17.0 | 16.5 |  |  |  |  |  |  |
| **65/F** | Days | 7 | 17 | 42 | 57 | 72 | 92 | 112 | 132 | 152 | 182 |  |  |  |  |
|  | cPDL | 4.3 | 8.5 | 11.5 | 12.9 | 15.4 | 17.6 | 19.9 | 21.0 | 22.1 | 21.8 |  |  |  |  |
| **72/M** | Days | 7 | 17 | 27 | 42 | 62 | 82 | 102 | 132 | 162 | 192 |  |  |  |  |
|  | cPDL | 3.6 | 6.5 | 9.8 | 12.0 | 14.0 | 16.1 | 17.9 | 20.2 | 20.8 | 17.9 |  |  |  |  |
| **71/F** | Days | 7 | 22 | 42 | 62 | 82 | 102 | 132 | 162 | 192 | 222 |  |  |  |  |
|  | cPDL | 3.6 | 5.9 | 8.5 | 9.0 | 10.1 | 12.6 | 14.7 | 16.8 | 17.3 | 15.8 |  |  |  |  |

M, male; F, female; P, passage number; Days, days in culture; cPDL, cumulative population doubling level of nucleus pulposus chondrocytes.
