# Supplementary material for: Relationship between Initial Telomere Length, Initial Telomerase Activity, Age, and Replicative Capacity of Nucleus Pulposus Chondrocytes in Human Intervertebral Discs: What Is a Predictor of Replicative Potential?
Source: PLoS One. 2015 Dec 3;10(12):e0144177. doi: 10.1371/journal.pone.0144177 (PMC4669191; doi:10.1371/journal.pone.0144177)
Supplement: S2 Table — (DOCX) [file pone.0144177.s002.docx]

**S2 Table. Telomere length and telomerase activity of nucleus pulposus chondrocytes obtained from study subjects.**

| **Age/Sex** | **TL and TA** | **P1** | **P3** | **P5** | **P7** | **P9** | **P11** |
| --- | --- | --- | --- | --- | --- | --- | --- |
| **39/M** | TL (Kb) | 22.0 (0.64) | 14.7 (0.48) | 12.7 (0.34) | 10.3 (0.11) | 8.0 (0.5) | 6.3 (0.24) |
|  | TA (RTA) | 31.5 (2.4) | 13.8 (0.99) | 13.4 (0.85) | 10.3 (1.27) | 5.6 (1.69) | 4.5 (0.56) |
| **32/M** | TL (Kb) | 13.4 (0.23) | 11.0 (0.42) | 10.6 (0.36) | 9.4 (0.04) | 8.0 (0.17) |  |
|  | TA (RTA) | 29.9 (0.84) | 17.9 (1.83) | 17.2 (2.4) | 8.9 (0.84) | 7.8 (1.13) |  |
| **37/M** | TL (Kb) | 11.1 (0.15) | 10.2 (0.09) | 7.3 (0.17) | 6.1 (0.1) | 5.9 (0.11) |  |
|  | TA (RTA) | 24.3 (1.55) | 17.2 (1.13) | 6.8 (1.84) | 5.8 (0.56) | 2.9 (2.43) |  |
| **44/M** | TL (Kb) | 16.0 (0.45) | 14.2 (0.69) | 14.2 (0.71) | 13.4 (0.32) |  |  |
|  | TA (RTA) | 15.9 (1.83) | 5.7 (0.77) | 4.7 (1.6) | 3.1 (0.83) |  |  |
| **44/F** | TL (Kb) | 14.2 (0.18) | 13.4 (0.23) | 12.6 (0.35) |  |  |  |
|  | TA (RTA) | 15.1 (1.02) | 13.5 (2.19) | 3.5 (0.78) |  |  |  |
| **41/M** | TL (Kb) | 11.0 (0.15) | 9.4 (0.07) | 8.2 (0.24) | 7.1 (0.17) |  |  |
|  | TA (RTA) | 16.3 (0.84) | 7.3 (0.89) | 6.4 (1.6) | 4.2 (0.51) |  |  |
| **58/M** | TL (Kb) | 10.3 (0.12) | 8.0 (0.11) | 7.1 (0.04) | 6.9 (0.1) |  |  |
|  | TA (RTA) | 14.1 (1.37) | 10.6 (3.16) | 3.4 (1.17) | 3.1 (0.41) |  |  |
| **52/M** | TL (Kb) | 12.6 (0.62) | 12.2 (0.66) | 11.0 (0.34) | 9.8 (0.19) | 9.0 (0.11) |  |
|  | TA (RTA) | 14.4 (3.38) | 13.8 (1.49) | 3.5 (0.83) | 4.0 (0.77) | 3.4 (0.69) |  |
| **58/F** | TL (Kb) | 17.7 (0.35) | 11.8 (1.18) | 10.2 (0.26) | 8.3 (0.14) |  |  |
|  | TA (RTA) | 10.2 (1.24) | 7.2 (2.34) | 9.0 (0.72) | 5.4 (0.56) |  |  |
| **66/M** | TL (Kb) | 7.0 (0.38) | 5.9 (0.29) | 5.7 (0.28) | 4.9 (0.19) |  |  |
|  | TA (RTA) | 13.1 (2.77) | 9.0 (1.71) | 2.1 (1.4) | 1.6 (0.72) |  |  |
| **63/M** | TL (Kb) | 12.6 (0.12) | 10.2 (0.11) | 9.4 (0.11) |  |  |  |
|  | TA (RTA) | 17.0 (2.27) | 9.1 (1.15) | 8.8 (0.22) |  |  |  |
| **65/F** | TL (Kb) | 19.7 (0.28) | 17.3 (0.12) | 12.6 (0.25) | 11.4 (0.06) | 9.8 (0.11) |  |
|  | TA (RTA) | 9.9 (1.04) | 6.9 (0.96) | 6 (1.58) | 4.8 (3.14) | 2.7 (0.53) |  |
| **72/M** | TL (Kb) | 11.0 (0.32) | 7.7 (0.09) | 7.1 (0.11) | 6.4 (0.14) |  |  |
|  | TA (RTA) | 9.3 (0.24) | 7.4 (1.55) | 7.7 (0.68) | 1.2 (0.12) |  |  |
| **71/F** | TL (Kb) | 18.1 (0.2) | 11.8 (0.17) | 9.4 (0.12) | 8.0 (0.08) |  |  |
|  | TA (RTA) | 22.3 (0.62) | 13.5 (5.09) | 3.5 (0.89) | 1.1 |  |  |

M, male; F, female; P, passage number; TL, telomere length of nucleus pulposus chondrocytes; TA, telomerase activity of nucleus pulposus chondrocytes; kb, kilobases; RTA, relative telomerase activity. Data are mean (SD) from two assays.
